# Small-Molecule Antioxidant Proteome-Shields in *Deinococcus radiodurans*

**DOI:** 10.1371/journal.pone.0012570

**Published:** 2010-09-03

**Authors:** Michael J. Daly, Elena K. Gaidamakova, Vera Y. Matrosova, Juliann G. Kiang, Risaku Fukumoto, Duck-Yeon Lee, Nancy B. Wehr, Gabriela A. Viteri, Barbara S. Berlett, Rodney L. Levine

**Affiliations:** 1 Department of Pathology, Uniformed Services University of the Health Sciences, Bethesda, Maryland, United States of America; 2 Armed Forces Radiobiology Research Institute, Uniformed Services University of the Health Sciences, Bethesda, Maryland, United States of America; 3 Laboratory of Biochemistry, National Heart, Lung, and Blood Institute, National Institutes of Health, Bethesda, Maryland, United States of America; National Institutes of Health, United States of America

## Abstract

For *Deinococcus radiodurans* and other bacteria which are extremely resistant to ionizing radiation, ultraviolet radiation, and desiccation, a mechanistic link exists between resistance, manganese accumulation, and protein protection. We show that ultrafiltered, protein-free preparations of *D. radiodurans* cell extracts prevent protein oxidation at massive doses of ionizing radiation. In contrast, ultrafiltrates from ionizing radiation-sensitive bacteria were not protective. The *D. radiodurans* ultrafiltrate was enriched in Mn, phosphate, nucleosides and bases, and peptides. When reconstituted *in vitro* at concentrations approximating those in the *D. radiodurans* cytosol, peptides interacted synergistically with Mn^2+^ and orthophosphate, and preserved the activity of large, multimeric enzymes exposed to 50,000 Gy, conditions which obliterated DNA. When applied *ex vivo*, the *D. radiodurans* ultrafiltrate protected *Escherichia coli* cells and human Jurkat T cells from extreme cellular insults caused by ionizing radiation. By establishing that Mn^2+^-metabolite complexes of *D. radiodurans* specifically protect proteins against indirect damage caused by gamma-rays delivered in vast doses, our findings provide the basis for a new approach to radioprotection and insight into how surplus Mn budgets in cells combat reactive oxygen species.

## Introduction

Unrepaired DNA double-strand breaks (DSBs) are generally the cause of ionizing radiation-induced cell-killing, as shown, for example, by the greatly increased radiosensitivity of specific repair-deficient mutants. However, the great variation in radiosensitivity among bacterial species correlates not with initial damage to DNA but rather with the susceptibility of their proteins to radiation-induced oxidation [1], [2]. For example, 90% of *Shewanella oneidensis* cells, which are hypersensitive to γ-ray-induced protein oxidation, are killed by doses of γ-rays (70 Gy) which cause less than one DSB per haploid genome [3], [4]. In contrast, proteins in extremely radiation resistant bacteria are highly protected from oxidation and the cells can survive hundreds of DSBs caused by ionizing radiation [1], [2], [5].

Remarkably, for bacteria spanning the limits of ionizing radiation resistance [3], [6], for human cells [7], for archaea, yeast, animals and viruses [5], [8], [9], the lesion-yields for DSBs, the most severe form of DNA damage in irradiated cells, are very similar and fall within a narrow range (0.002–0.006 DSB/Gy/Mbp per haploid genome). In contrast, the amount of protein damage in irradiated cells is strongly influenced by their antioxidant status, where yields of radiation-induced protein oxidation can be 100 times greater in sensitive bacteria than in resistant bacteria [5]. We have hypothesized that naturally sensitive bacteria are killed by ionizing radiation mainly owing to the susceptibility of their repair proteins to oxidative inactivation, which could render even minor DNA damage lethal. In contrast, manganese complexes in extremely resistant bacteria may prevent oxidative protein damage, which could protect the activity of enzymes, and thereby greatly increase the efficiency of DNA repair [5]. This exploratory study is the first to examine the nature of radioprotective Mn complexes in *D. radiodurans*.

In the 1940s, Walter M. Dale demonstrated that enzymes in aqueous solution could be inactivated by small doses of X-rays (10 Gy), mediated by the indirect effects of reactive molecular species derived from the ionization of water [10]–[12]. The possibility that resistance of cells to ionizing radiation could be increased, specifically by protecting proteins, was supported by studies which showed that the radiosensitivity of an enzyme is not a fixed entity but a variable, where inactivation could be prevented by adding an enzyme's substrate or other small organic compounds [10], [11]. In the 1960s, a low-molecular-weight (<15 kDa), protein-free extract capable of protecting sensitive bacteria against the lethal effects of ionizing radiation was prepared from *Deinococcus radiodurans* [13], a vegetative bacterium which represents life's utmost limit for ionizing radiation resistance, capable of surviving 12,000 Gy [14]–[16]. Yet, the active components of the extract and the cellular molecules they protected were not identified [13]. The concordance of this history of results with recent work demonstrating that proteins, but not DNA, in *Deinococcus* bacteria are extraordinarily resistant to ionizing radiation and desiccation damage [1], [3], [5], [17] led to this study.

Our strategy for elucidating the chemical protective mechanisms utilized by *D. radiodurans* was to identify inorganic and organic constituents in protein-free cell extracts of *D. radiodurans* which were over-represented compared to protein-free cell extracts of ionizing radiation-sensitive bacteria. Of the small molecules which were enriched in *D. radiodurans* protein-free cell extracts, peptides were by far the most abundant. At physiologically relevant concentrations, reconstituted mixtures of peptides, Mn^2+^ and orthophosphate bestowed extraordinary levels of radiation resistance on purified enzymes but did not significantly protect DNA. Collectively, our findings resolve how, after exposure to huge doses of γ-rays, or to months of desiccation in a desert, *D. radiodurans* cells retain sufficient protein activity to repair their DNA.

## Results

### Bacterial ultrafiltrates

**Figure 1:**
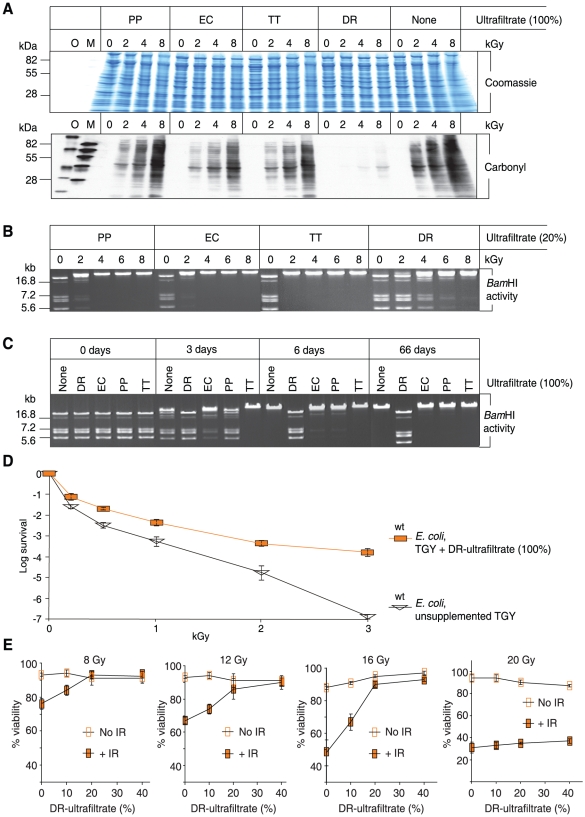
*In vitro* and *ex vivo* protection by DR-ultrafiltrate. (**A**) DR-ultrafiltrate prevents protein oxidation. The indicated ultrafiltrates were mixed with purified *E. coli* proteins and irradiated to the indicated doses of μ-radiation (kGy). Proteins were then separated by polyacrylamide gel electrophoresis and visualized by Coomassie staining. Duplicate gels were subjected to Western blot carbonyl analysis, which reveals the presence (black) or absence (no signal) of protein oxidation. PP, *P. putida*; EC, *E. coli*; TT, *T. thermophilus*; and DR, *D. radiodurans*. O and M, size-standards. (**B**) DR-ultrafiltrate preserves the activity of an irradiated enzyme. *Bam*HI was irradiated in the indicated ultrafiltrates, then incubated with μ-DNA and subjected to agarose gel electrophoresis. (**C**) DR-ultrafiltrate preserves the activity of a desiccated enzyme. *Bam*HI was desiccated from the indicated ultrafiltrates and stored in a desiccator for the indicated times, and then assayed for residual activity as in panel B. (**D**) DR-ultrafiltrate protects *E. coli*. Wild-type *E. coli* (MM1925) cells were grown in TGY medium supplemented with DR-ultrafiltrate and irradiated without change of broth to the indicated doses, then recovered on TGY medium. Colony forming unit (CFU) survival assays were in triplicate for each dose, with standard deviations shown. (**E**) DR-ultrafiltrate protects human Jurkat T cells. DR-ultrafiltrate was added to the growth medium 1 day before irradiation. The viability of irradiated cells was determined by trypan blue staining 2 days after irradiation. Viability assays were in triplicate, with standard deviations shown.

The four model bacteria investigated here have been the subjects of extensive bioinformatic and experimental comparative analyses [1], [3], [14], [18]. They are the protagonists of our ‘Death by Protein Damage’ model of ionizing radiation toxicity [5]. For *D. radiodurans* (DR) and the ionizing radiation-sensitive bacteria *Pseudomonas putida* (PP), *Escherichia coli* (EC) and *Thermus thermophilus* (TT), aqueous-phase extracts of cell homogenates were first subjected to ultracentrifugation, and then to ultrafiltration. Ultracentrifugation removed proteins and other macromolecules, and peptides which were greater than 1 kDa; as a precaution, the ultracentrifuged supernatants were ultrafiltrated to remove any contaminating molecules (>3 kDa) released from the pellets during collection of the supernatants. When PP-, EC- or TT-ultrafiltrates were mixed with proteins purified from *E. coli* and exposed to γ-radiation, high levels of protein oxidation were detected by Western blot carbonyl analysis (Figure 1A); carbonyl groups are the most widely used marker of severe protein oxidation [19]. In contrast, the DR-ultrafiltrate was extremely protective against ionizing radiation-induced protein carbonylation (Figure 1A). We note here that the effects of bacterial ultrafiltrates on protein oxidation by ionizing radiation *in vitro* parallel the levels of protein oxidation observed for the corresponding bacterial species irradiated *in vivo* [1]. We also tested the ability of the four ultrafiltrates to protect the activity of the restriction endonuclease *Bam*HI, which is readily inactivated in aerobic aqueous solutions by reactive oxygen species (ROS) generated by 150 Gy [1]. The DR-ultrafiltrate preserved the activity of *Bam*HI exposed aerobically to 4 kGy, but PP-, EC- or TT-ultrafiltrates did not (Figure 1B). When desiccated from DR-ultrafiltrate, *Bam*HI survived at least 66 days, but when desiccated from PP-, EC- or TT-ultrafiltrates, *Bam*HI activity was lost after six days (Figure 1C). We note here that desiccation dose-response relationships for bacterial protein oxidation and survival observed *in vivo* coincide with these enzyme activity results [17]. Thus, the DR-ultrafiltrate rendered the ROS-sensitive *Bam*HI highly resistant to ionizing radiation and desiccation in the same range as *D. radiodurans* survival [3]. Extreme resistance to desiccation, ultraviolet (UV) radiation and ionizing radiation are mechanistically coupled in diverse organisms [5], [8], [15], [17], [20]. However, a chemical basis of resistance to these oxidizing conditions in Mn-accumulating bacteria has not yet been investigated.

Consistent with the report of a ‘radioresistant factor’ in protein-free extracts of *D. radiodurans* [13], the DR-ultrafiltrate was radioprotective of *E. coli* (Figure 1D). Radioprotection by DR-ultrafiltrate was not limited to bacteria. Protection extended to human lymphoblastoid Jurkat T cells cultured *in vitro*, where exposure to 16 Gy typically kills greater than 50% of cells within 2 days [21] (Figure 1E and S1). In a concentration-dependent manner, treatment of Jurkat T cells with DR-ultrafiltrate fully preserved their viability up to 16 Gy (Figure 1E and S1), a dose which causes approximately 280 DNA DSBs per haploid genome in human cells [7]. We infer that components of the DR-ultrafiltrate were taken up by *E. coli* and Jurkat T cells, but this has not been investigated.

### Small-molecule profiling of the bacterial ultrafiltrates

**Figure 2:**
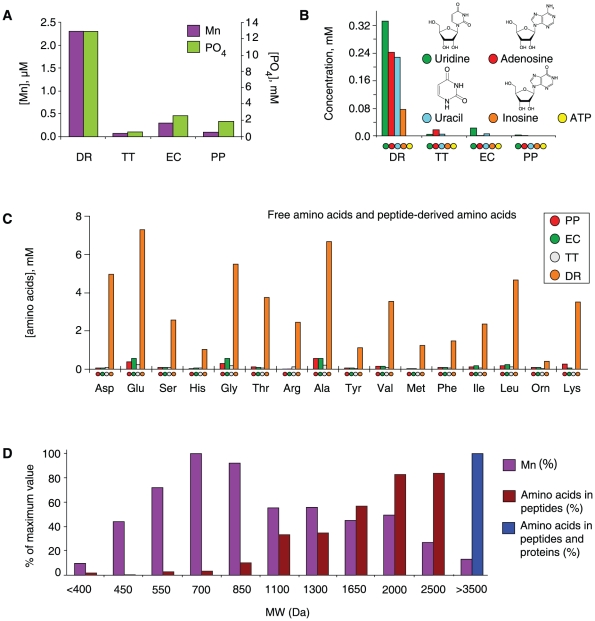
Composition of the DR-ultrafiltrate. (**A**) Manganese (Mn) and phosphate (PO_4_) concentrations in bacterial ultrafiltrates (100%). (**B**) Nucleoside and base (Ns/Nb) concentrations in bacterial ultrafiltrates (100%) (Table S1). (**C**) Sum of free amino acids and those in peptide linkage in bacterial ultrafiltrates (100%) after acid hydrolysis. The total amino acid concentration of the DR-ultrafiltrate is 53 mM, of which 97% are in peptides (see also Figure S2). (**D**) Mn-complexes. The distribution of Mn bound to small molecules and peptides in aqueous-phase extracts of *D. radiodurans* homogenate was determined by size exclusion chromatography. The cell homogenate was prepared from cells disrupted in the presence of protease inhibitors. Note, gel filtration causes a significant dilution of the original sample, so the measured concentration in the fractions does not reflect the undiluted concentration in the sample. The 100% value for amino acids is 330 µM and that for Mn is 3.8 µM. As any unbound Mn or free amino acid-bound Mn would have eluted late in the chromatographic analysis, we concluded that Mn was bound to peptides. Proteins and large peptides >3,500 Da eluted together at the exclusion volume of the column; average molecular weight of an amino acid in peptide linkage is 115 Da.

The chemical constituents of the DR-ultrafiltrate which were identified and whose concentrations were elevated compared to the PP-, EC- and TT-ultrafiltrates include Mn (Figure 2A), phosphate (Figure 2A), uridine, adenosine and uracil (Figure 2B and Table S1), and amino acids and peptides (Figure 2C and S2). The accumulation of Mn is a hallmark of all *Deinococcus* bacteria [5], [17], [20], and it is established that *D. radiodurans* exposed to ionizing radiation or UV radiation produces an intracellular pool of nucleotides which are subsequently converted to nucleosides and then exported [22]. Whereas iron concentrations in *D. radiodurans* are comparable to ionizing radiation-sensitive bacteria, *D. radiodurans* exhibits Mn concentrations which are 15–150 times greater than radiosensitive bacteria [3]. Under our growth conditions, the total cellular Mn concentration in *D. radiodurans* was approximately 200 µM (Table 1), although localized Mn concentrations in *D. radiodurans* can be significantly higher [1]. Most of the cytosolic Fe (83%) in *D. radiodurans* was bound to proteins, which were precipitated by trichloroacetic acid (TCA), but most of the cytosolic Mn (72%) was resistant to TCA-precipitation (Table 1). In the aqueous-phase extract of *D. radiodurans* homogenate, which was used to prepare DR-ultrafiltrate, Mn was bound to small molecules (<3 kDa), which included peptides (Figure 2D). Others have established that Mn^2+^ ions can form antioxidant complexes with various inorganic and organic compounds. Mn^2+^ and orthophosphate, which do not significantly scavenge hydroxyl radicals (HO^•^) [1] (Figure S3A), form complexes which catalytically remove superoxide (O_2_^•−^) via a disproportionation mechanism [23], [24]; and amino acids and peptides, which scavenge HO^•^ very efficiently, form complexes with Mn^2+^ which catalytically decompose hydrogen peroxide (H_2_O_2_) [25].

**Table 1:** Manganese and iron concentrations in cells and their extracts^A^.

**Whole cells** B **^,^** C	**Mn (**µ**M)**	**Fe (**µ**M)**
*D. radiodurans*	192	200
*E. coli*	13.1	118
**TCA extract of cell homogenate** B **^,^** D	**Mn (**µ**M)**	**Fe (**µ**M)**
*D. radiodurans*	138	34.8
*E. coli*	11.7	30.8
**Phosphate extract of cell homogenate** B **^,^** E	**Mn (**µ**M)**	**Fe (**µ**M)**
*D. radiodurans*	152	124
*E. coli*	11.1	112
**Ultrafiltrate** F	**Mn (**µ**M)**	**Fe (**µ**M)**
*D. radiodurans*	2.3	8.1
*E. coli*	0.3	1.2
*P. putida*	0.1	2.2
*T. thermophilus*	0.1	2.0

(Footnotes to Table 1 A–F).

A Concentration values are the average of triplicate analyses using a Perkin Elmer model 4100ZL atomic absorption spectrometer.

B Cells were grown in a 20-L fermentor under previously described conditions [56] to the stationary-phase, quick-frozen in liquid nitrogen, and stored at −80°C. At the time of analysis, the cells were thawed and washed three times with phosphate buffer, pH 7.4 at 4°C.

C Cells were boiled in 5% nitric acid to extract metals. Metal analyses were by atomic absorption spectroscopy (AAS). Whereas cellular Fe concentrations in *D. radiodurans* were comparable to *E. coli*, *D. radiodurans* exhibited Mn concentrations which were 15 times greater than *E. coli*.

D Cells were resuspended (1∶1) in 20% trichloroacetic acid (TCA), disrupted and then centrifuged (12,000×*g*, 1h), and the supernatants analyzed by AAS. As the water content of cells approximates 1ml/g cell, the TCA concentration after disruption was 10%; the 10% TCA precipitated all proteins, but not small molecules. 83% of the cytosolic Fe in *D. radiodurans* TCA homogenates was precipitated, but most of the cytosolic Mn (72%) escaped TCA-precipitation.

E Cells were resuspended in phosphate buffer, pH 7.4, disrupted and then centrifuged (12,000×*g*, 1h), and the aqueous-phase extracts analyzed by AAS. Cells were completely disrupted by the homogenization procedure.

F Cells were grown as batch cultures in TGY, harvested, washed and resuspended in de-ionized H_2_O. The cell suspensions were homogenized and then ultracentrifuged (190,000×*g*, 69 h). The supernatants were passed through 3 kDa filters and analyzed by AAS. Most of the Mn and Fe in *D. radiodurans* homogenate were lost during ultracentrifugation, which removed molecules greater than ∼1,000 Da [57].

**Figure 3:**
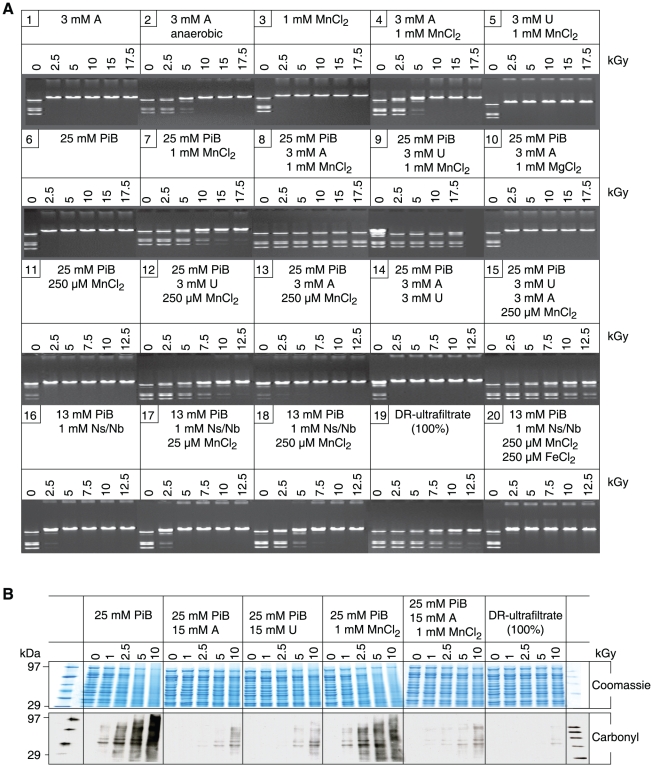
Radioprotection by mixtures of Mn^2+^, orthophosphate, nucleosides and bases. (**A**) *Bam*HI activity. *Bam*HI was irradiated in the indicated mixtures and then assayed for residual activity as in Figure 1B. Abbreviations: PiB, potassium phosphate buffer, pH 7.4; A, adenosine; U, uridine; Ns/Nb, nucleosides and bases (1 mM; see Table S1 for the Ns/Nb added). Irradiations were under aerobic conditions unless stated otherwise (gel 2). (**B**) Nucleosides prevent protein oxidation. Proteins purified from *E. coli* were mixed with adenosine (A) or uridine (U), phosphate buffer (PiB), pH 7.4 and Mn^2+^, and irradiated to the indicated doses (kGy). The irradiated proteins were separated by polyacrylamide gel electrophoresis and visualized by Coomassie staining as in Figure 1A. Note, the ability of the DR-ultrafiltrate to prevent *in vitro* ionizing radiation-induced protein carbonylation corresponds to the preservation of stainable (Coomassie) banding.

We previously demonstrated that *Bam*HI is more sensitive to O_2_^•−^ than HO^•^ generated by ionizing radiation; under aerobic conditions, *Bam*HI was inactivated by 150 Gy, but survived 800 Gy under anaerobic conditions [1]. The DR-ultrafiltrate was enriched in adenosine and other nucleosides and bases (Figure 2B and Table S1), which scavenge HO^•^ efficiently but do not react with O_2_^•−^ [26]. Figure 3A (gel 1) shows that *Bam*HI did not survive 2.5 kGy when irradiated aerobically in 3 mM adenosine, but survived 5 kGy when irradiated anaerobically in 3 mM adenosine (Figure 3A, gel 2). Thus, the radioprotective effects of limiting O_2_^•−^- and HO^•^-mediated damage on the enzyme's activity were synergistic.

### Multifactorial mode of enzyme protection under ionizing radiation

**Figure 4:**
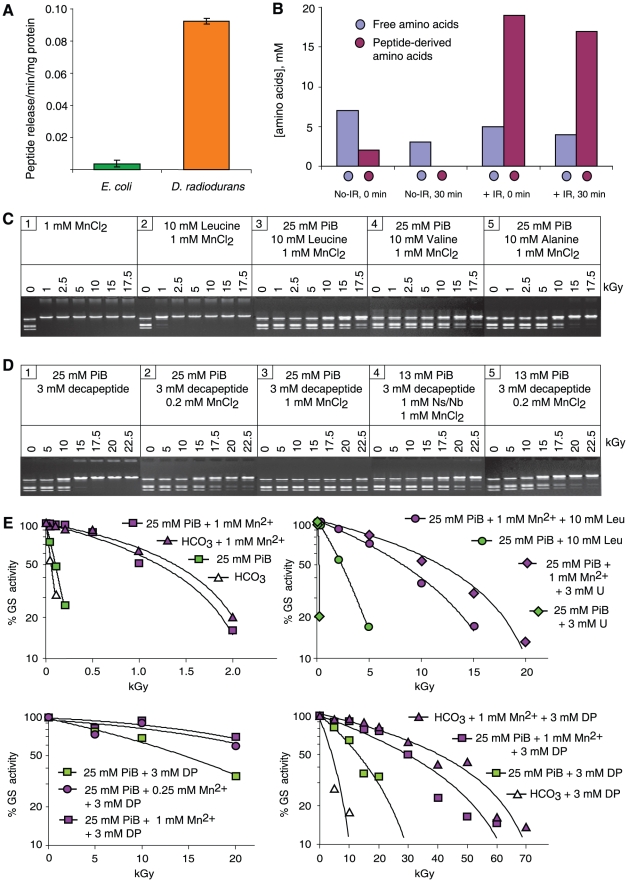
Role of amino acids and peptides in ionizing radiation resistance. (**A**) Cytosolic protease activities in *E. coli* and *D. radiodurans*. (**B**) Cytosolic distribution and concentration of amino acids in *D. radiodurans*. No-IR, non-irradiated control cells held in 25 mM phosphate buffer, pH 7.4 on ice, then washed and held in 25 mM phosphate buffer, pH 7.4 (32°C) for 0 or 30 min. +IR, cells irradiated to 7 kGy in 25 mM phosphate buffer, pH 7.4 on ice, then washed and held in 25 mM phosphate buffer, pH 7.4 (32°C) for 0 or 30 min. Cells were harvested, resuspended in 20% TCA, and broken open. Aliquots of neutralized supernatant were analyzed for free amino acid and peptide-derived amino acid content. (**C**) Radioprotection of *Bam*HI by amino acids. PiB, potassium phosphate buffer, pH 7.4. (**D**) Radioprotection of *Bam*HI by the decapeptide (H-Asp-Glu-His-Gly-Thr-Ala-Val-Met-Leu-Lys-OH; 1,261 Da). Ns/Nb, nucleosides and bases (1 mM; see Table S1 for the Ns/Nb added). (**E**) Radioprotection of glutamine synthetase (GS) by Mn^2+^ and leucine (Leu), uridine (U), or the decapeptide (DP) in potassium phosphate buffer (PiB), pH 7.4 or sodium bicarbonate buffer (HCO_3_), pH 7.4. Adenosine could not be evaluated because it is an allosteric inhibitor of glutamine synthetase.

Since the DR-ultrafiltrate was enriched in Mn^2+^, inorganic phosphate, nucleosides and bases (Figure 2A and 2B), we tested the radioprotective properties of their mixtures on *Bam*HI. As most of the Mn in *D. radiodurans* homogenate was lost during ultracentrifugation (bound to molecules >1 kDa; Table 1), we tested a range of Mn^2+^ concentrations (0.2–1 mM) which matched total intracellular Mn concentrations reported for *D. radiodurans* (see below). The orthophosphate concentration we used *in vitro* was 25 mM, which is physiologically relevant to many cell-types, including eukaryotes [27]. Individually, adenosine, Mn^2+^, and phosphate buffer did not protect *Bam*HI from 2.5 kGy under aerobic conditions (Figure 3A, gels 1, 3 and 6). Together, Mn^2+^ and phosphate buffer protected *Bam*HI to 10 kGy (Figure 3A, gel 7); and an equivalent mixture which also contained adenosine or uridine protected *Bam*HI to 17.5 kGy (Figure 3A, gels 8 and 9). The substrate of *Bam*HI is DNA, and to determine whether or not radioprotection extended to other classes of enzymes, we tested purified glutamine synthetase. This dodecameric enzyme plays a central role in intermediary metabolism, and is very sensitive to oxidative inactivation by multiple species of ROS [28]. When glutamine synthetase was irradiated in phosphate buffer alone, or phosphate buffer and uridine, 50% activity survived 0.1 kGy (Figure 4E). When irradiated in Mn^2+^ and phosphate buffer, 50% glutamine synthetase activity survived 1 kGy (Figure 4E). However, when glutamine synthetase was irradiated in an equivalent mixture of phosphate buffer and uridine which contained Mn^2+^, 50% activity survived 10 kGy (Figure 4E). The presence of Mn^2+^ was paramount since Mg^2+^, Ca^2+^, Fe^2+^, Ni^2+^, Cu^2+^ and Zn^2+^ had no protective effect when combined with nucleosides and phosphate buffer (Figure 3A, gel 10, and Figure S3B). *In vitro*, removal of O_2_^•−^ by Mn^2+^ is dependent on a threshold concentration of Mn^2+^ [1], [23], [24]. When the concentration of Mn^2+^ in phosphate buffer was lowered from 1 mM to 250 µM, radioprotection of *Bam*HI was lost, but protection was restored by the addition of uridine (Figure 3A, gels 11–15) or analogues containing two carbonyl oxygen groups (C = O) separated by one (N3)H group (Figure S3C), a configuration which forms stable Mn^2+^ complexes when deprotonated [29]. We note that radioprotection of *Bam*HI by uridine was enhanced by the addition of adenosine when Mn^2+^ and phosphate buffer were present (Figure 3A, gels 11–15), raising the possibility of a role for adenosine-uridine interactions.

When the most abundant free nucleosides and bases detected in the DR-ultrafiltrate (Table S1) were combined with Mn^2+^ and phosphate buffer, the mixtures were not as radioprotective of *Bam*HI as the DR-ultrafiltrate (Figure 3A, gels 16–19). Moreover, the capacity of the DR-ultrafiltrate to prevent *in vitro* ionizing radiation-induced protein damage exceeded mixtures of nucleosides and Mn-phosphate (Figure 3B, Coomassie and Carbonyl). Thus, the DR-ultrafiltrate contains radioprotectants beyond Mn^2+^, phosphate, nucleosides and bases.

### Role of amino acids and peptides in resistance

Stadtman and colleagues discovered and characterized an unexpected property of complexes consisting of Mn^2+^ and amino acids or peptides, namely their ability to catalytically scavenge ROS [25]. We noted above that DR-ultrafiltrate is highly enriched in amino acids and peptides in comparison to the PP-, EC- or TT-ultrafiltrates (Figure 2C), and chromatographic analysis indicated that Mn was bound to peptides in aqueous-phase extracts of the *D. radiodurans* homogenates used to prepare DR-ultrafiltrate (Figure 2D). We determined peptide concentrations by comparing the amino acid content with and without acid hydrolysis. The latter detects only free amino acids while the hydrolyzed sample detects the sum of free amino acids and those in peptide linkage. *D. radiodurans* displays very high intracellular protease activities (Figure 4A), and we considered the possibility that peptides detected in the DR-ultrafiltrate were artifacts generated during processing; indeed, proteins in *D. radiodurans* homogenates were being degraded when homogenates were prepared in phosphate buffer at 0°C. To prevent *ex situ* protein degradation, we examined aqueous-phase extracts of *D. radiodurans* homogenates prepared from cells disrupted in the presence of 20% TCA, which completely inactivates enzymes and precipitates all proteins released upon cell lysis, but does not remove small molecules or their complexes. Based on cells disrupted in the presence of 20% TCA, the total cytosolic concentration of amino acids (free and peptide-derived) in non-irradiated *D. radiodurans* was 9 mM, of which 22% were in peptide linkage (2 mM) (Figure 4B). For *D. radiodurans* exposed to 7 kGy, the total cytosolic concentration of amino acids was 24 mM, of which 79% were in peptide linkage (19 mM) (Figure 4B). Exposure of *D. radiodurans* to 7 kGy increased the cytosolic concentration of amino acids in peptide linkage by approximately 10 times, which we attribute to ionizing radiation-induced proteolysis (Figure 4A). In contrast, the total amino acid concentration of the DR-ultrafiltrate was 53 mM (Figure 2C), of which 97% were in peptide linkage (51 mM). Thus, *ex situ* protein degradation in homogenates prepared in phosphate buffer was substantial, but the presence of 20% TCA during disruption of non-irradiated *D. radiodurans* cells reduced the level of peptides by 96%. As the free amino acid and peptide-derived amino acid contents of irradiated or non-irradiated *D. radiodurans* did not increase when the cells were held in phosphate buffer at 32°C for 30 minutes (Figure 4B); and as the concentrations of peptide-derived amino acids in the TCA-homogenates of *D. radiodurans* did not change when incubated for one hour at room temperature (data not shown), we are confident that the TCA inactivated any proteases in the extract.

**Figure 5:**
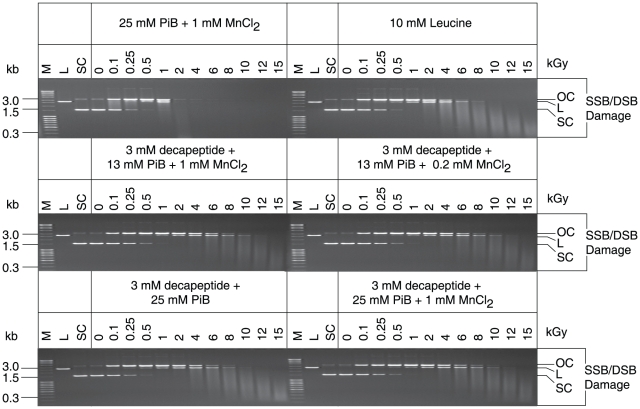
The HO^•^-scavenging properties of Mn^2+^, orthophosphate, leucine and the decapeptide (H-Asp-Glu-His-Gly-Thr-Ala-Val-Met-Leu-Lys-OH). Structural forms of the plasmid (pUC19): OC, open circular; L, linear; and SC, super-coiled. SSB, single-strand break; DSB, double-strand break. M, DNA size marker; PiB, phosphate buffer, pH 7.4. In the absence of HO^•^-scavenging agents, approximately 80% of ionizing radiation-induced damage to purified DNA in aqueous solution is caused by HO^•^, where one SSB in a SC circular plasmid molecule yields an OC form [30]. In contrast to DNA damage, 3 mM decapeptide, 25 mM phosphate buffer, pH 7.4, and 1 mM Mn^2+^ preserved the activity of enzymes exposed to 50 kGy (Figure 4E).

We tested the radioprotective properties of the amino acids Leu, Val and Ala, and a synthetic decapeptide (H-Asp-Glu-His-Gly-Thr-Ala-Val-Met-Leu-Lys-OH) whose composition matched that of some of the most abundant amino acids in the hydrolyzed DR-ultrafiltrate (Figure 2C). In the enzyme radioprotection assays (Figure 4C, D and E), the amino acid and peptide concentrations were in the same range as those in *D. radiodurans* homogenates prepared in 20% TCA. At 10 mM, Leu or Val were highly radioprotective of *Bam*HI when combined with 1 mM Mn^2+^ in 25 mM phosphate buffer (Figure 4C). As most peptides in *D. radiodurans* homogenate were 7 to 22 amino acids in length (Figure 2D), the cytosolic concentration of peptides in irradiated *D. radiodurans* was approximately 1–3 mM (19 mM/22 to 19 mM/7) (Figure 4B). In comparison, the peptide concentration in DR-ultrafiltrate was between 2–8 mM (Figure 2C). At 3 mM, the decapeptide protected the activity of irradiated *Bam*HI to 10 kGy when combined with 25 mM phosphate buffer (Figure 4D, gel 1); when 1 mM Mn^2+^ was added to the decapeptide-phosphate buffer mixture, *Bam*HI survived greater than 22.5 kGy (Figure 4D, gels 3–4), which far exceeded radioprotection by Mn^2+^-phosphate buffer alone (Figure 3A, gel 7). In parallel studies which tested the absolute limits of enzyme radioprotection, a combination of the 3 mM decapeptide in phosphate buffer or bicarbonate buffer (Figure 4E) with 0.25–1 mM Mn^2+^ preserved 20–50% activity of glutamine synthetase exposed to 50 kGy. In stark contrast, DNA incubated in equivalent mixtures of Mn^2+^, decapeptide and phosphate buffer was obliterated by 12 kGy (Figure 5); and enzymes irradiated in phosphate buffer alone were inactivated by less than 0.5 kGy (Figure 4E).

### Radioprotection of cells

**Figure 6:**
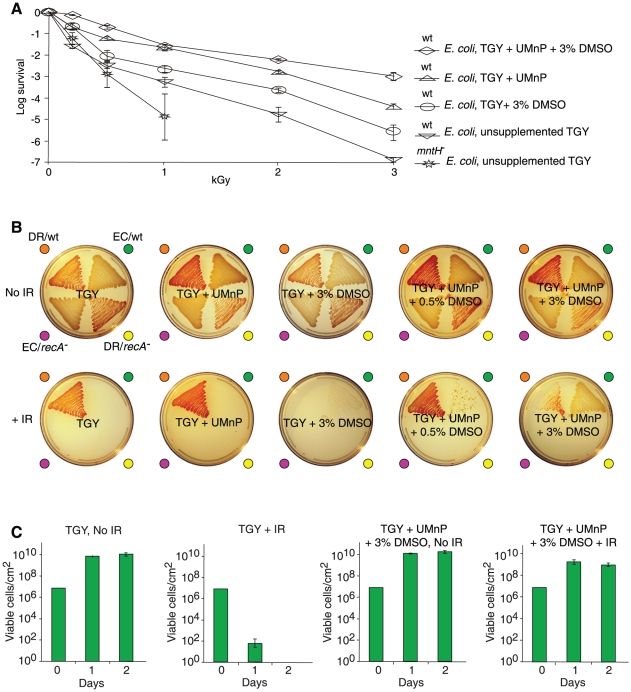
Radioprotection of *E. coli*. (**A**) Survival of *E. coli* exposed to acute ionizing radiation (IR). Cells were grown in, irradiated in, and recovered on the indicated medium (see also Figure S4B). wt, wild-type (MM1925); *mntH* ^−^, isogenic Mn-transport mutant (MM2115); UMnP contained 3 mM uridine/1 µM Mn^2+^/13 mM phosphate buffer, pH 7.4; DMSO, dimethyl sulfoxide. As the HO^•^-scavenger uridine is a good growth substrate for *E. coli* (Figure S4A) and is not accumulated by the cells, we included DMSO, which *E. coli* does not metabolize (Figure S4A). Standard deviations shown. (**B**) Growth of *E. coli* under high-level chronic μ-radiation on solid medium (TGY). No IR, non-irradiated control plates incubated for 2 days at 25°C; +IR, plates incubated under 42 Gy/hour at the same temperature for the same time. Strain abbreviations: DR/wt, wild-type *D. radiodurans*; EC/wt, wild-type *E. coli* (MM1925); DR/*recA* ^−^ (rec30), *D. radiodurans* DNA repair mutant; EC/*recA* ^−^ (DH10B), *E. coli* DNA repair mutant. Each agar sector was inoculated with 1×10^7^ cells (see also Figure S4C). (**C**) Quantification of *E. coli* growth under chronic ionizing radiation. Cells on 4 cm^2^ agar sectors corresponding to those in panel B were harvested at 1 and 2 days after incubation under 42 Gy/hour at 25°C. Each agar sector was inoculated with 1×10^7^ cells. The number of viable cells per sector after 1 or 2 days was enumerated in triplicate, with standard deviations shown. Note, *E. coli* cells harvested from TGY+UMnP+3% DMSO+IR did not grow when transferred to non-supplemented TGY+IR.

We tested an *ex vivo* radioprotection strategy based on our approach to preserving the activity of irradiated enzymes, by treating *E. coli* with reagents which protected proteins *in vitro*. Added individually or in combination, the radioprotective properties of Mn^2+^, phosphate buffer, uridine and dimethyl sulfoxide (DMSO; a specific HO^•^-scavenger which is not metabolized by *E. coli*; Figure S4A) [30] were determined using *E. coli* grown in TGY, an undefined rich medium which contains 200 nM Mn [3] (Figure 6A and S4B). When TGY was supplemented individually with Mn^2+^, uridine, DMSO or phosphate buffer, the resistance of *E. coli* exposed to 3 kGy was increased by 0, 10, 50 and 800 times, respectively (Figure 6A and S4B). When these agents were combined at concentrations applied individually, the survival of *E. coli* exposed to 3 kGy was increased by 10,000 times (Figure 6A). Thus, the radioprotective synergistic effects manifested between HO^•^-scavenging agents and catalytic O_2_^•−^-scavenging agents on the survival of acutely irradiated *E. coli* (Figure 6A) mirrored those observed *in vitro* for irradiated enzymes (Figure 3A, 4C, 4D, and 4E).

Wild-type *E. coli* does not grow and is killed under high-level chronic γ-radiation (Figure 6B) [3]. We show that the same combination of HO^•^- and O_2_^•−^-scavenging agents which rendered *E. coli* resistant to high doses of acute ionizing radiation (Figure 6A), endowed *E. coli* with the ability to grow under high-level chronic irradiation. Under 42 Gy per hour, *E. coli* did not grow up on TGY; nor when TGY was supplemented with a mixture of uridine/Mn^2+^/phosphate buffer (UMnP); nor when TGY was supplemented with DMSO alone (Figure 6B). However, when equivalent mixtures of UMnP and DMSO were combined in TGY plates, wild-type *E. coli* displayed luxuriant growth under 42 Gy/hour (Figure 6B, 6C and S4C), a dose rate which kills all but the most ionizing radiation-resistant bacteria [3]. As for the radioprotective *ex vivo* effects of DR-ultrafiltrate (Figure 1D and 1E), we infer that components of the uridine/Mn^2+^/orthophosphate/DMSO mixtures were taken up by the cells, but this has not been investigated.

## Discussion

In the absence of radiation, eukaryotes and bacteria can survive large numbers of DSBs provided the cells functionally express a complement of DNA repair functions. Yeast can survive hundreds of endogenous DSBs generated during meiosis [31], bacteria can endure dozens of DSBs produced by the transient expression of cloned restriction endonucleases [32], and the rate of DSB production in normal human cells is estimated to be 50 per cell per cell cycle [33]. Yet, doses of ionizing radiation which cause equivalent numbers of DSBs are lethal to most cells. Recently, fresh insight into the reparability of DSBs was gained by comparisons of DNA and protein damage in irradiated bacteria which have very different antioxidant statuses and resistances. For a given dose of ionizing radiation, DSB lesion-yields were very similar, but protein oxidation lesion-yields were quantitatively related to survival [1], [2], [5].

Classical models of radiation toxicity assert that X-rays and γ-rays indiscriminately damage cellular macromolecules, principally by indirect effects mediated by HO^•^ [5], [34]. Water is the most abundant chemical found in living cells and the primary ROS which arise during the radiolysis of H_2_O are HO^•^ (H_2_O→HO^•^+H^+^ [proton]+e^−^_aq_ [hydrated electron]); H_2_O_2_ (2 HO^•^→H_2_O_2_); and O_2_^•−^ anions (O_2_+e^−^_aq_→O_2_^•−^) [5]. Whereas HO^•^ are extremely reactive and short-lived, O_2_^•−^ and H_2_O_2_ are relatively inert and long-lived [5], [35]. This, however, does not imply that HO^•^ will display greater toxicity [36]. The most consequential damage by O_2_^•−^ and H_2_O_2_ in cells is site-specific, to proteins which contain exposed iron-sulfur or haem groups [35], to proteins which contain cysteine residues, and to proteins containing cation-binding sites where an iron-catalyzed site-specific oxidation occurs [37].

Compared to most organisms, proteins in *D. radiodurans* are highly protected from ROS, but lose their resistance when purified from the cells [1]. In contrast, DNA in *D. radiodurans* is damaged with essentially the same dose dependence as in all prokaryotic and eukaryotic cells examined [3], [5], [8]. Notably, the multifactorial and synergistic modes of protection of irradiated enzymes by the reconstituted components of DR-ultrafiltrate (Figure 3A, 4C, 4D and 4E) were not manifested for purified DNA (Figure 5 and S3A). When orthophosphate (13 mM) (Figure 2A), Mn^2+^ (200 µM) (Table 1), and peptides (3 mM) (calculated from Figure 4B, see above) were combined *in vitro* at concentrations approximating those in *D. radiodurans*, the mixture preserved the activity of *Bam*HI and glutamine synthetase exposed to 17.5 kGy (Figure 4D, gels 3–5; and 4E), but did not significantly protect DNA (Figure 5). 17.5 kGy represents the outer limits of *D. radiodurans* survival and breaks its 4–8 haploid genomes per cell into 1,000–2,000 DSB fragments [3], [38]. Thus, protein protection mediated by small Mn complexes provides an explanation for the large shoulders in ionizing radiation dose-response curves of *D. radiodurans* survival which distinguish them from radiosensitive organisms [5], [8].

The key difference between naturally radiosensitive and radioresistant bacteria is that the latter appear to have developed chemical mechanisms for protecting their proteins. Based on whole-genome comparisons, there is a remarkable abundance in *D. radiodurans* of genes encoding catabolic enzymes including phosphatases, nucleases and proteases, which would be expected to give rise to the sorts of small molecules accumulated in the DR-ultrafiltrate. At this juncture, we believe that the peptide concentrations reported in DR-homogenate prepared in 20% TCA (Figure 4B) are physiologically relevant. However, this may not be the case for nucleosides and bases, which were only quantified in DR-ultrafiltrate (Figure 2B and Table S1). As noted above, it is known that *D. radiodurans* exposed to ionizing radiation produces an intracellular pool of nucleotides which are subsequently converted to nucleosides [22]. There is also a distinct possibility that other small molecules are accumulated in *D. radiodurans* which were not identified in this study. What we can say with confidence is that *D. radiodurans* has presented us with a highly protective chemical strategy against ionizing radiation and other oxidizing agents.

### Manganese in *D. radiodurans*

The first report of Mn accumulation in *D. radiodurans* was by Leibowitz and colleagues [39], who demonstrated that *D. radiodurans* contained approximately 100 times more Mn than *E. coli* when grown in a defined minimal medium (DMM). Using neutron activation analysis (NAA), they showed that *D. radiodurans* accumulated a total of ∼0.29×10^−18^ mol Mn/cell (∼1.8×10^5^ Mn atoms/cell; or ∼4 mM Mn, given a cell volume of 6.5×10^−2^µm^3^) when grown in DMM containing trace levels of Mn; when DMM was supplemented with 10 µM Mn^2+^, *D. radiodurans* accumulated a total of ∼1.6×10^−18^ mol Mn/cell (∼1×10^6^ Mn atoms/cell; or ∼22 mM Mn) [39]. In 2004, the NAA results were corroborated using inductively coupled plasma mass spectrometry [3]; and other studies showed that when *D. radiodurans* was incubated in minimal medium containing the radioisotope ^54^Mn, the cells accumulated approximately 3 mM Mn [3]. Recently, X-ray fluorescence (XRF) microspectroscopy revealed that Mn is distributed throughout *D. radiodurans* cells grown in TGY, but with regional intracellular Mn concentrations ranging from 0.4 to 3 mM [1]; the *D. radiodurans* cells studied by XRF, however, were desiccated, yielding higher Mn concentrations than would be present in hydrated cells. In contrast, much of the Fe in *D. radiodurans* was sequestered outside of the cytosol, in the septa of dividing cells [1]. Based on electron paramagnetic resonance (EPR) spectroscopy [3], [39] and X-ray-absorption near-edge structure (XANES) analyses [1], the dominant form of manganese in *D. radiodurans* cells is Mn^2+^, with no significant levels of Mn^3+^ detected. Using atomic absorption spectroscopy, we showed that *D. radiodurans* (grown in TGY) contains approximately 200 µM Mn, 72% of which was not bound to proteins in the corresponding homogenate (Table 1). Unfortunately, it was not possible to determine Mn speciation within cell extracts because low-molecular-weight Mn^2+^ complexes exchange their ligands very rapidly in solution, and standard analytical procedures that disrupt cells likely alter speciation [40].

### Role of metabolite accumulation in radiation resistance

The existence of chemical antioxidants in *D. radiodurans* which are based on common metabolites raises the possibility that a major route to radiation resistance and desiccation resistance is via metabolite regulation [41], where equivalent synergistic processes promoted by the accumulation of Mn^2+^ and small molecules may be acting similarly in other organisms [5]. *D. radiodurans* encodes an expanded family of proteases [14] which appear to be activated by irradiation (Figure 4B). At least 10 complete open reading frames with considerable sequence similarity to proteases of *Baccilus subtilis* have been identified in *D. radiodurans* [41], which are implicated herein as contributing to the pool of peptides needed to form antioxidant Mn^2+^ complexes. Moreover, wild-type *Deinococcus* species display unusual metabolic defects which could facilitate metabolite accumulation [41]. For example, *D. radiodurans* cannot utilize uridine or adenosine as carbon and energy sources (Figure S4A), which could explain the enrichment of these nucleosides in the DR-ultrafiltrate (Figure 2B). Other examples of metabolic deficiencies in wild-type *D. radiodurans* which could promote the accumulation of metabolites include: (i) *D. radiodurans* has a functional tricarboxylic acid (TCA) cycle but is unable to grow on a variety of non-fermentable carbon sources (*e.g.*, α-ketoglutarate, succinate, fumarate or malate) [41]. While such a metabolic configuration limits growth, it could promote the accumulation of TCA cycle products and their derivatives (*e.g.*, amino acids); and (ii) *D. radiodurans* has lost four genes in the biosynthetic pathway of nicotinamide adenine dinucleotide (NAD), a coenzyme which *D. radiodurans* requires for growth [41], [42]. The absence of enzymes in *D. radiodurans* which synthesize NAD could explain the accumulation of NAD precursors (nucleotides and their derivatives) in DR-ultrafiltrate. The metabolic configuration of *D. radiodurans* and its implications for ionizing radiation resistance have been described previously [41].

We demonstrate that wild-type *E. coli* can develop high levels of radioresistance based on supplementation alone (Figure 6). Resistance to high-level irradiation in naturally sensitive bacteria previously has only been achieved by serial selection of resistant mutants [43], [44]. Notably, as the mutants became more ionizing radiation-resistant, they progressively lost metabolic functions and displayed heterotrophic nutritional modes which resembled those of *Deinococcus* species [41]. The benefits of Mn accumulation in cells which are unable to amass small molecules may be limited. By replacing Fe^2+^ and other divalent cations (*e.g.*, Mg^2+^ and Cu^2+^) with Mn^2+^ as mononuclear cofactors in enzymes, active sites would be protected from oxidative damage [45] but might remain vulnerable to primary ROS at other sites in the absence of proximal ligands which activate Mn^2+^ (*e.g.*, orthophosphate, bicarbonate, amino acids, peptides and nucleosides) (Figure 3A, 4C, 4D and 4E). Thus, any of a number of mutations which cause the loss of metabolic functions could lead to metabolite accumulation and radiation resistance, provided the cells express Mn transport systems.

As the survival of irradiated enzymes and their hosts rests squarely on preventing both site-specific (O_2_^•−^ and H_2_O_2_) and non-specific (HO^•^) forms of ROS damage, the accumulation of Mn^2+^ together with certain organic metabolites may represent a widespread strategy for combating oxidative stress. For example, dormant spores of *Bacillus* species accumulate high levels of both Mn and dipicolinic acid, as well as a large depot of small proteins (∼70 amino acid residues in length) [46]; cyanobacteria accumulate Mn^2+^ and nonreducing disaccharides [47]; and numerous other environmentally robust Mn-rich microorganisms accumulate mycosporin-like amino acids [48]; and in mitochondria and chloroplasts, where organellar Mn budgets appear to exceed the demands of their enzymes [49], [50], Mn might form antioxidant complexes as in radiation resistant bacteria.

### Application prospects

Since the 1960s, the goal of exporting the radioprotective processes of *D. radiodurans* outside of the host cell for practical purposes has eluded researchers [5], [13]–[16]. *E. coli* and other bacteria which were previously considered far too sensitive to grow under high-level chronic ionizing radiation now emerge as prospective candidates for bioremediation at highly radioactive sites [51] (Figure 6B and 6C). The finding that the treatment of human Jurkat T cells with DR-ultrafiltrate rescued them from γ-ray exposures (16 Gy) which cause ∼560 DSBs per diploid cell [7] (Figure 1E and S1) demonstrates that metabolic interventions at the cellular level may be a powerful approach to fight oxidative stress in animal cells during irradiation or aging [52]. Another tangible application of our approach may be the preparation of ionizing radiation-sterilized whole-bacterial cell, whole-virus, or protein vaccines with only nominal loss in immunogenicity. Our finding that mixtures of Mn^2+^ and the decapeptide in bicarbonate buffer protected approximately 50% activity of the dodecameric glutamine synthetase (622 kDa) at 50 kGy (Figure 4E), supports that complex quaternary protein structures in aqueous solution can be preserved at doses of ionizing radiation which destroy nucleic acids (Figure 5 and S3A) [3], [15], [16], [39]. Others have shown that bacteria exposed to 8 kGy are able to trigger long-lasting immunity [53]. However, the anticipated levels of ionizing radiation required to inactivate bacteria without any risk of infection would render vaccines with no immunogenicity due to oxidation of their antigenic determinants [53]. Similar drawbacks apply to viruses, which require even greater ionizing radiation doses than bacteria for sterilization. Our approach to protecting proteins could be applied to preparing irradiated vaccines, where the epitopes of cells or viruses treated with Mn-peptide complexes in orthophosphate or bicarbonate buffer would be expected to survive doses of ionizing radiation which irreversibly inactivate their genomes.

### Conclusion

Recent studies implicate proteins as critical early targets in irradiated cells [1], [2], [54]. DNA repair-proficient bacteria which are unable to protect their proteins from ionizing radiation-induced ROS succumb to relatively minor DNA damage [1], [4], [5]. While impaired DNA DSB repair provides the best available correlation with radiation-induced cell-killing, protection of proteins in radiation resistant bacteria by Mn-dependent chemical antioxidants generally provides an explanation for extreme resistance without invoking the need for novel repair pathways or unusual forms of DNA packaging [5].

Although Mn^2+^ is most commonly associated with its role as a catalytic cofactor of proteins [27], the majority of cellular Mn in *D. radiodurans* appears to exist as small Mn^2+^ complexes (Figure 2C). Moreover, intracellular Mn^2+^ speciation within yeast has recently been probed through measurements of ^1^H and ^31^P electron-nuclear double resonance (ENDOR) signal intensities [27], which support an important *in vivo* role for the orthophosphate complex of Mn^2+^ (Mn^2+^-Pi) in cellular resistance to oxidative stress. Those studies support that Mn^2+^-Pi, but not Mn^2+^-polyphosphate or Mn^2+^-pyrophosphate, can compensate for the loss of superoxide dismutase enzymes and that Mn^2+^-Pi truly serves as a cellular antioxidant [27]. In *D. radiodurans*, the accumulation of Mn^2+^-Pi and peptides is strongly implicated in protecting the proteome from oxidation. We propose that the great efficiency of DNA repair in *D. radiodurans* is based on Mn^2+^-Pi-metabolite complexes, which specifically protect cytosolic proteins against ionizing radiation- and desiccation-induced oxidation and thereby preserve the function of enzymes needed to repair DNA and survive.

A recent mathematical model of radiogenic oxidative stress is consistent with our data and can potentially be generalized to other organisms and lower radiation doses [55]. Our studies indicate that supplementation of bacteria and human cells with mixtures of peptides, nucleosides, Mn^2+^ and orthophosphate, which are enriched in DR-ultrafiltrate, is a major route to radiation resistance mediated by protein protection. Thus, our findings could come to affect approaches to pre-exposure and post-exposure interventions in diverse settings of oxidative stress management.

## Materials and Methods

### Bacterial ultrafiltrates

The DR- (ATCC BAA-816), PP- (ATCC 47054), EC- (MG1655) and TT- (ATCC BAA-163) ultrafiltrates (Figure 1A, 1B, 1C, 2, 3, and S2) were prepared from bacteria grown as batch cultures (8×1.5 L each) in TGY medium (1% Bacto Tryptone, 0.5% Bacto Yeast Extract and 0.1% glucose) [1], [3] to an optical density of 0.9 (log-phase) determined at 600 nm. For the large-scale production of DR-ultrafiltrate used in radioprotection studies of *E. coli* and Jurkat T cells (Figure 1D, 1E and S1), *D. radiodurans* (ATCC BAA-816) was grown under optimized conditions using a 20-L fermentor [56]. Harvested cells were washed twice in distilled and de-ionized H_2_O (ddH_2_O), centrifuged at 2,000×*g* for 15 min at 4°C, and broken open by passage through a French Press (20,000 lb/in^2^) as described previously [1]. Upon lysis, crude cell extracts (homogenates) were centrifuged at 12,000×*g* (1 h, 4°C) yielding an aqueous-phase which contained soluble proteins and small molecules, and a pellet of insoluble cell debris. Using the Coomassie (Bradford) assay (BioRad, Hercules, California, USA), the supernatants were standardized for concentration on a protein-basis [1] (18.6 mg/ml) by dilution with ddH_2_O. The adjusted supernatants were ultracentrifuged at 190,000×*g* (69 h, 4°C), which removed molecules greater than 1,000 Da [57]. The ultracentrifuged supernatants were subjected to filtration through 3 kDa Centriplus Centrifugal Filter Devices (YM-3) (Millipore Corporation, Bedford, Massachusetts, USA) at room temperature for 3–4 hours. The ultrafiltrates were then boiled for 40 min, concentrated 5 times in a SpeedVac concentrator (Savant, GMI, Inc., Ramsey, Minnesota, USA) at room temperature, aliquoted, and stored at −80°C.

### Assay for oxidized proteins

Proteins purified from an *E. coli* (MG1655) homogenate were used as the substrate for carbonyl analyses reported in (Figure 1A and 3B). Proteins were prepared from *E. coli* grown in TGY to OD_600_ 0.9. Cells were broken open by passage through a French Press (20,000 lb/in^2^), and the soluble protein fraction was recovered from the cell homogenate by centrifugation at 12,000×*g* (1 h, 4°C) and quantified for concentration using the Bradford assay [1]. The proteins were stored at −80°C as 35 mg/ml stocks in 150 µl aliquots, which were used once and then discarded. Before irradiation, a stock of *E. coli* proteins was diluted in ddH_2_O and 5×concentrated DR-, PP-, EC- or TT-ultrafiltrate preparations, yielding mixtures with 4.4 mg/ml of *E. coli* protein in 1×ultrafiltrate (100%) (Figure 1A). For a given *E. coli* protein-ultrafiltrate sample (100 µl), irradiations were on ice under aerobic conditions. At the indicated ionizing radiation doses (Figure 1A), 20 µl samples were removed and stored on ice until the time-course of irradiation was completed. The carbonyl groups in the *E. coli* protein samples were then derivatized to 2,4-dinitrophenylhydrazone by reaction with 2,4-dinitrophenylhydrazine for 15 min in 3% (w/v) sodium dodecyl sulfate [1]. Derivatized samples and size standards were processed for carbonyl analysis as described previously [1].

### Irradiations

*E. coli* protein-ultrafiltrate samples (Figure 1A and 3B) and *E. coli* protein-nucleoside-phosphate samples (Figure 3B) were irradiated in air on ice with ^60^Co at 4.2 kGy/h. Irradiations of *Bam*HI (Figure 1B, 3A, 4C, 4D, S3B and S3C) and glutamine synthetase (Figure 4E) were in air on ice with ^60^Co at 4.2 kGy/h and 13.3 kGy/h, respectively. Acute irradiations of *E. coli* cells (Figure 1D, 6A, and S4B) were in air on ice with ^60^Co at 3.7 kGy/h. Acute irradiations of Jurkat T cells (Figure 1E and S1) were at room temperature with ^60^Co at 36 Gy/h. Growth of *D. radiodurans* and *E. coli* under high-level chronic irradiation (Figure 6B, 6C, and S4C) was at 42 Gy/h (^137^Cs).

### Assays for enzyme activity and DNA damage

The chemical agents identified in the DR-ultrafiltrate (Figure 2A, 2B, 2C, 4B, and S2; and Table S1) were reconstituted *in vitro* using reagents from Sigma Chemical Company (St. Louis, Missouri, USA). The synthetic decapeptide (H-Asp-Glu-His-Gly-Thr-Ala-Val-Met-Leu-Lys-OH) was obtained from Elim Biopharmaceuticals, Inc. Hayward, California, USA, and was authenticated at NHLBI by high performance liquid chromatography-mass spectrometry (HPLC-MS). Dilutions of reagents were made with ddH_2_O. Post-irradiation activity of *Bam*HI was determined as described previously [1]. Briefly, *Bam*HI (3,000 U/µl) (without BSA) (New England Biolabs, Ipswich, Massachusetts, USA) was diluted in the specified bacterial ultrafiltrate to 0.54 U/µl (Figure 1B) or diluted in the various reagent-mixtures to 3.6 U/µl (Figure 3A, 4C, 4D, S3B, S3C); typically, 200 µl of the *Bam*HI mixtures were irradiated aerobically. For the anaerobic *Bam*HI irradiation (Figure 3A, gel 2), 0.5 ml samples were first purged with ultra-high purity argon (99.999%) and then irradiated in sealed tubes [1]. Following irradiation, 20 µl of each ionizing radiation-treated *Bam*HI sample were assayed for residual endonuclease activity in separate reaction mixtures (final volume, 30 µl) containing 125 ng μ-phage DNA, 50 mM NaCl, 10 mM Tris-HCl (pH 7.9), 10 mM MgCl_2_, and 1 mM dithiothreitol (New England Biolabs). *Bam*HI/μ DNA mixtures were incubated for 1.25 h at 37°C, followed by agarose (0.8%) gel electrophoresis [1] (AGE). Post-desiccation activity of *Bam*HI (Figure 1C) was determined as follows. *Bam*HI (3,000 U/µl) (without BSA) (New England Biolabs) was diluted in the specified bacterial ultrafiltrates (Figure 1C), yielding pre-desiccation samples with 20 U/µl of *Bam*HI in 1×ultrafiltrate (Figure 1C). Pre-desiccation samples (8 µl) were transferred to 0.5 ml Eppendorf tubes, which were placed open in an aerobic desiccation chamber over anhydrous calcium sulfate (W. A. Hammond Drierite Company, Ltd., Xenia, Ohio, USA). The desiccation chamber was hermetically sealed and stored at room temperature. At the indicated times, the dried samples were dissolved in 8 µl 1×DR-ultrafiltrate (Figure 1C) and assayed for residual endonuclease activity using μ-phage DNA and AGE as for irradiated *Bam*HI samples (see above). Glutamine synthetase (GS) (40 µg/ml) purification from *E. coli*, and GS activity measurements post-irradiation were as described previously [28]; each trial presented in Figure 4E was repeated twice. For DNA damage assays, supercoiled pUC19 DNA (1 µg/µl) (New England Biolabs) was diluted (1/25) in the specified reagent mixtures (Figure 5 and S3A).

### Survival assays for *E. coli* and human Jurkat T cells

Wild-type *E. coli* (K12, MM1925) [58] (Figure 1D, 6, and S4) or its isogenic *mntH* ^−^ (Mn-transport) mutant (MM2115) [58] (Figure 6A) were inoculated into TGY liquid medium supplemented or not with the indicated reagents; note, growth and recovery of MM2115 was in the presence of kanamycin (50 µg/ml). The bacteria were grown at 37°C until a culture reached OD_600_ 0.9 (∼5×10^8^ colony forming units (CFUs)/ml). For liquid cultures exposed to acute doses of ionizing radiation (^60^Co), cell survival was determined by serial dilution and CFU analysis [3], with three biological replicates performed for each trial (Figure 1D, 6A, and S4B). The ability of bacteria to grow under high-level chronic irradiation was tested on unsupplemented and supplemented TGY medium solidified with 1.5% Bacto agar (Figure 6B, 6C, and S4C). For a given strain, the bacteria (1×10^7^ cells) were seeded as lawns onto 4 cm^2^ sectors, and incubated for 2 days (Figure 6B). The *recA* ^−^ mutant of *E. coli* (DH10B) and *recA* ^−^ mutant of *D. radiodurans* (rec30) were as described previously [59]. In parallel studies, growth of wild-type *E. coli* (MM1925) under 42 Gy/h was quantified (Figure 6C), where each trial consisted of a single plate with a 4 cm^2^ sector inoculated with 1×10^7^ cells. Cells from a sector were harvested and enumerated by CFU assay after 24 h, and a second batch of inoculated plates was enumerated after 48 h (Figure 6C). Note, inoculations of ‘TGY+IR’ sectors (4 cm^2^) with ∼1×10^8^ *E. coli* (MM1925) cells sporadically gave rise to 1–3 colonies per sector, derived from spontaneous ionizing radiation-resistant mutants within a lawn which did not grow. TGY sectors which gave rise to colonies under ionizing radiation were not enumerated for lawn-growth. Thus, sectors (Figure 6B and 6C) were inoculated with 1×10^7^ cells. Low-frequency spontaneous ionizing radiation resistant *E. coli* mutants precluded quantification in liquid culture under 42 Gy/h. Human Jurkat T cells (ATCC TIB-152) were inoculated at 1×10^6^ cells/ml and grown in 75 cm^2^ tissue culture flasks (Costar, Cambridge, Massachusetts, USA) containing RPMI 1640 medium supplemented with 0.03% glutamine, 4.5 g/l glucose, 25 mM HEPES, 10% fetal bovine serum, penicillin (50 µg/ml) and streptomycin (50 µg/ml) (Gibco/BRL, Gaithersburg, Maryland, USA). The cells were incubated at 37°C in a 5% CO_2_-incubator and fed every 3–4 days. Twenty-four hours before irradiation (^60^Co), the cells were treated with DR-ultrafiltrate (Figure 1E and S1). For each acute dose of ionizing radiation, the Jurkat T cell trials (2 ml with 1×10^6^ cells/ml) were performed in triplicate in 6-well microtiter plates. After irradiation, the microtiter plates were returned to the 5% CO_2_-incubator for the specified time (1–3 days), and cell viability (Figure 1E and S1) was determined with trypan blue [60]. For each trial, 50 µl of Jurkat T cells were mixed with 50 µl of 4% trypan blue; 20 µl of the cell suspensions were enumerated for viable cells by hemocytometer counts (Figure 1E and S1).

### Composition of bacterial extracts

The chemical composition of the DR-, PP-, EC- and TT-ultrafiltrates (Figure 2A, 2B, 2C, and S2; and Table S1) prepared from cells grown in batch culture (see above) was determined as follows: Mn and Fe on a Perkin Elmer model 4100ZL atomic absorption spectrometer; inorganic phosphate by the malachite green assay [61]; bases, nucleosides and nucleotides by HPLC [62]; and amino acids by pre-column derivatization [63] as implemented by Agilent Technologies [64]. HPLC analysis without acid hydrolysis gave the free amino acid content while analysis following acid hydrolysis gave the free and peptide bound content (Figure 2C and S2). The analyses of cell homogenates which were not ultracentrifuged (Figure 2D and 4B; and Table 1) were on cells grown in a 20-L fermentor [56]. Cells were grown to the stationary phase, quick-frozen in liquid nitrogen, then stored at −80°C. At the time of analysis, the cells were thawed and washed three times with 50 mM phosphate buffer, pH 7.4 at 4°C. To determine the size-distribution of small molecules and Mn in *D. radiodurans* homogenates (Figure 2D), the cells were resuspended in 50 mM phosphate buffer, pH 7.4 containing a protease inhibitor cocktail (Roche “Complete Solution for Protease Inhibition”, 1 mini-tablet per 10 ml buffer; Roche, Basel, Switzerland). The cells were then broken open by a One Shot cell homogenizer (Constant Systems Limited, Daventry, England, UK) at 40,000 lb/in^2^. The homogenates were centrifuged at 12,000×*g* (1 h, 4°C), and the molecular weight distribution of Mn, peptides and proteins in the soluble extracts was determined by gel filtration on a 25×1.7 cm column packed with Bio-Gel P4 (Figure 2D). Fractioned aliquots of the soluble extract were subjected to acid hydrolysis and analyzed as described above; and the Mn content of fractions was determined by atomic absorption spectroscopy. In studies which determined the cytosolic amino acid and peptide concentrations in soluble extracts of *D. radiodurans* homogenates (Figure 4B), the cells were resuspended (1∶1) in 20% trichloroacetic acid (TCA), disrupted at 40,000 lb/in^2^ (One Shot Cell Disrupter) and centrifuged at 12,000×*g* (1 h, 4°C). The supernatants were analyzed for amino acids with or without hydrolysis (Figure 4B; data sets are the averages of 2 independent trials), and also for Mn and Fe concentrations (Perkin Elmer model 4100ZL) (Table 1). Protease activities in cell homogenates of *E. coli* (MG1655) and *D. radiodurans* (ATCC BAA-816) (Figure 4A) were determined as follows. *E. coli* and *D. radiodurans* were each grown in a 20-L fermentor [56], washed in 50 mM phosphate buffer, pH 7.4, and broken open with a One Shot Cell Disrupter at 40,000 lb/in^2^ (Constant Systems, Ltd.) as described above, yielding soluble extracts. Quantification of protein in the soluble extracts was by the bicinchoninic acid assay (Thermo Scientific, Pierce Protein Research Products, Rockford Illinois, USA). Proteolytic activity was assayed for 20 min at 40°C using azocasein as substrate [65]. The reaction was stopped by precipitating the protein with an equal volume of 20% TCA, vortexing 5 s, incubating 5 min at room temperature, and then centrifuging 20,000×*g* (5 min, 4°C). The absorbances were determined at 366 nm, and the peptide release calculated; the assay was repeated 4 times on different aliquots.

## Supporting Information

Figure S1 DR-ultrafiltrate preserves the viability of irradiated Jurkat T cells. Jurkat T cells in liquid medium were adjusted to 40% DR-ultrafiltrate 24 h before acute irradiation (16 Gy). The viability of irradiated cells was determined by trypan blue staining after 1, 2 and 3 days. Viability assays were in triplicate, with standard deviations shown.
(0.65 MB EPS)

Figure S2 Free amino acid composition of PP-, EC-, TT- and DR-ultrafiltrates. Strain abbreviations as in Figure 1A. The free amino acid concentration of the DR-ultrafiltrate is 1.4 mM. Note, the sum of free amino acids and those in peptide linkage in the PP-, EC-, TT- and DR-ultrafiltrates after acid hydrolysis is presented in Figure 2C. The total amino acid concentration of the DR-ultrafiltrate is 53 mM (Figure 2C), of which 97% are in peptides.
(0.72 MB EPS)

Figure S3 Mixtures of Mn^2+^ and orthophosphate, and nucleosides and bases protect irradiated enzymes but not DNA. (A) The HO^•^-scavenging properties of Mn^2+^, phosphate buffer (PiB, pH 7.4), nucleosides and bases (Ns/Nb) (see Table S1 for Ns/Nb added) were tested. pUC19 DNA damage assays and abbreviations as in Figure 5. (B) Radioprotective properties of divalent metals on *Bam*HI. Assay conditions as in Figure 3A, gel 10. (C) Radioprotective properties of nucleosides, bases and nucleotides on *Bam*HI when Mn^2+^ is limited. Assay conditions as in Figure 3A, gels 11–15. PiB, phosphate buffer, pH 7.4.
(3.33 MB EPS)

Figure S4 Radioprotection of *E. coli*. (A) *E. coli* can grow on nucleosides but *D. radiodurans* cannot. Strain abbreviations as in Figure 1A. Defined minimal medium (DMM) [3] was supplemented with 1% (128 mM) DMSO, 10 mM fructose, 10 mM uridine, or 10 mM adenosine. Cells were pre-grown in DMM with 22 mM fructose for 2 days and then diluted to OD_600_ 0.1 in the indicated medium. Incubations (PP, 32°C; EC, 37°C; TT, 65°C; DR, 32°C) were in triplicate for 70 h, with standard deviations shown. (B) Survival of wild-type (wt) *E. coli* (MM1925) exposed to acute ionizing radiation (kGy). Cells were grown in, irradiated in, and recovered on the indicated medium: UMnP contained 3 mM uridine/1 µM Mn^2+^/13 mM phosphate buffer, pH 7.4; DMSO, dimethyl sulfoxide; PiB, phosphate buffer, pH 7.4. (C) Co-incubation of *E. coli* and *D. radiodurans* under high-level chronic ionizing radiation (42 Gy/h). *E. coli* (MM1925) and *D. radiodurans* were inoculated onto the plates as concentric rings. Within plate 4, the trefoil pattern (red stripes) corresponds to the location of 3 agar segments which were excised and exchanged with the corresponding agar segments from plate 1 immediately after inoculation. Plate 1 (control) was incubated for 3 days at 25°C without ionizing radiation (IR). Plates 2–4 were incubated for 3 days at 25°C under 42 Gy/h. Abbreviations as in panel B. Color key: green, *E. coli* (EC); orange, *D. radiodurans* (DR). Since growth of *D. radiodurans* on TGY under 42 Gy/h is inhibited by 3% DMSO (Figure 6B), this approach (panel C) allowed us to demonstrate growth of *D. radiodurans* (red-pigmented) and *E. coli* (non-pigmented) on the same plate under 42 Gy/h.
(6.81 MB EPS)

Table S1 Concentration of nucleosides (Ns), bases (Nb) and nucleotides (Nt) in the bacterial ultrafiltrates (100%).
(0.05 MB DOC)
