# Supplementary material for: Small-Molecule Antioxidant Proteome-Shields in Deinococcus radiodurans
Source: PLoS One. 2010 Sep 3;5(9):e12570. doi: 10.1371/journal.pone.0012570 (PMC2933237; doi:10.1371/journal.pone.0012570)
Supplement: Table S1 — Concentration of nucleosides (Ns), bases (Nb) and nucleotides (Nt) in the bacterial ultrafiltrates (100%). (0.05 MB DOC) [file pone.0012570.s005.doc]

**Table S1. Concentration of nucleosides (Ns), bases (Nb) and nucleotides (Nt) in the bacterial ultrafiltrates (100%)**

| Ns/Nb/Nt | ^b^DR-ultrafiltrate | ^b^EC-ultrafiltrate | ^b^PP-ultrafiltrate | ^b^TT-ultrafiltrate |
| --- | --- | --- | --- | --- |
| ^a^**Uridine, mM** | **0.33** | 0.023 | 0.003 | 0.004 |
| ^a^**Adenosine, mM** | **0.24** | 0.001 | 0.001 | 0.018 |
| ^a^**Uracil, mM** | **0.23** | 0.005 | 0.000 | 0.005 |
| ^a^**Deoxyuridine, mM** | **0.078** | 0.000 | 0.000 | 0.001 |
| ^a^**Inosine, mM** | **0.076** | 0.000 | 0.000 | 0.001 |
| ^a^**Thymidine, mM** | **0.037** | 0.001 | 0.001 | 0.000 |
| ^a^**Guanosine, mM** | **0.016** | 0.000 | 0.000 | 0.002 |
| Adenine, mM | 0.013 | 0.014 | 0.007 | 0.003 |
| AMP, mM | 0.003 | 0.005 | 0.035 | 0.000 |
| Deoxycytidine, mM | 0.002 | 0.011 | 0.000 | 0.000 |
| GTP, mM | 0.002 | 0.001 | 0.001 | 0.002 |
| Cytidine, mM | 0.001 | 0.000 | 0.000 | 0.000 |
| Deoxyguanosine, mM | 0.000 | 0.000 | 0.000 | 0.000 |
| Deoxyadenosine, mM | 0.000 | 0.000 | 0.000 | 0.000 |
| NADP, mM | 0.000 | 0.000 | 0.000 | 0.000 |
| ATP, mM | 0.000 | 0.002 | 0.000 | 0.001 |
| *Total, mM* | *1.028* | *0.063* | *0.048* | *0.036* |

^a^At the indicated concentrations present in the ultrafiltrate, the *D. radiodurans* Ns/Nb in bold were added to irradiated enzyme preparations reported in Figure 3A and 4D, and pUC19 samples in Supplementary Figure 3A. The total concentration of the indicated Ns/Nb in the DR-ultrafiltrate was ~1 mM.

^b^Strain abbreviations as in Figure 1A.
